# Assessment of drinking water quality using Water Quality Index and synthetic pollution index in urban areas of mega city Lahore: a GIS-based approach

**DOI:** 10.1038/s41598-024-63296-1

**Published:** 2024-06-11

**Authors:** Maria Latif, Nimra Nasir, Rab Nawaz, Iqra Nasim, Khawar Sultan, Muhammad Atif Irshad, Ali Irfan, Turki M. Dawoud, Youssouf Ali Younous, Zulkifl Ahmed, Mohammed Bourhia

**Affiliations:** 1 Department of Environmental Sciences, The University of Lahore, Lahore, 54000 Pakistan; 2 Faculty of Engineering and Quantity Surveying, INTI International University, 71800 Nilai, Negeri Sembilan Malaysia; 3 Department of Chemistry, Government College University Faisalabad, Faisalabad, 38000 Pakistan; 4 Botany and Microbiology Department, College of Science, King Saud University, P.O. Box 2455, 11451 Riyadh, Saudi Arabia; 5 Evangelical College, BP 1200, N’Djamena, Chad; 6 College of Resource and Civil Engineering, Northeast University, Shenyang, China; 7 Laboratory of Biotechnology and Natural Resources Valorization, Faculty of Sciences, Ibn Zohr University, 80060 Agadir, Morocco

**Keywords:** Water contamination, Water Quality Index, Drinking water quality, Physiochemical parameters, Public health, Ecology, Environmental sciences

## Abstract

The aim of the present study was to assess the drinking water quality in the selected urban areas of Lahore and to comprehend the public health status by addressing the basic drinking water quality parameters. Total 50 tap water samples were collected from groundwater in the two selected areas of district Lahore i.e., Gulshan-e-Ravi (site 1) and Samanabad (site 2). Water samples were analyzed in the laboratory to elucidate physico-chemical parameters including pH, turbidity, temperature, total dissolved solids (TDS), electrical conductivity (EC), dissolved oxygen (DO), total hardness, magnesium hardness, and calcium hardness. These physico-chemical parameters were used to examine the Water Quality Index (WQI) and Synthetic Pollution Index (SPI) in order to characterize the water quality. Results of th selected physico-chemical parameters were compared with World Health Organization (WHO) guidelines to determine the quality of drinking water. A GIS-based approach was used for mapping water quality, WQI, and SPI. Results of the present study revealed that the average value of temperature, pH, and DO of both study sites were within the WHO guidelines of 23.5 °C, 7.7, and 6.9 mg/L, respectively. The TDS level of site 1 was 192.56 mg/L (within WHO guidelines) and whereas, in site 2 it was found 612.84 mg/L (higher than WHO guidelines), respectively. Calcium hardness of site 1 and site 2 was observed within the range from 25.04 to 65.732 mg/L but, magnesium hardness values were higher than WHO guidelines. The major reason for poor water quality is old, worn-out water supply pipelines and improper waste disposal in the selected areas. The average WQI was found as 59.66 for site 1 and 77.30 for site 2. Results showed that the quality of the water was classified as “poor” for site 1 and “very poor “ for site 2. There is a need to address the problem of poor water quality and also raise the public awareness about the quality of drinking water and its associated health impacts.

## Introduction

One of the most important necessities for preserving human health is the availability of clean drinking water. Water is the most prevalent compound on the Earth surface and is a renewable resource that is necessary for life to exist. Unfortunately, the water will become increasingly scarcer as a result of population growth, urbanization, and climate change^1^. Industrial effluents are recognized as major contaminants in groundwater and sewer waters. Industries that release waste and effluents into water bodies without treatment have affected the environment, endangered human health, and disrupted aquatic ecosystems. Groundwater is contaminated with heavy metals and other pollutants due to the widespread dumping of industrial waste and effluents into water bodies without any kind of treatment or filtration. For the efficient treatment of water bodies, there are a variety of treatment technologies such as improved oxidation processes, phytoremediation, and nano-remediation^2–4^. Water resources in developing nations, such as Pakistan, are contaminated due to a number of industrial and human activities. People rely on heavily contaminated sources, like shallow wells and boreholes, for drinking water due to the insufficient water supply, which creates serious health hazards. In addition, these polluted water sources are unfit for residential use, which makes it even harder for communities to get access to clean and safe drinking water^5^.

Recent technological developments, such as chemical composition of pipelines, can occasionally result in the pollution of drinking water with biological, physical, and chemical pollutants. Improper or inadequate supply of drinking water poses a serious threat to public health. The water quality in most of the Pakistani cities is deteriorating rapidly^6^. Human health is greatly affected by the lack of access to sanitary facilities and clean water. Every year, the use of tainted water and inadequate sanitation systems cause around 2.2 million deaths among the population in poorer nations. The Sustainable Development Goals (SDGs) estimate that 1.2 billion people worldwide lack access to even the most basic services related to water. Remarkably, eight out of ten people live in rural regions without access to basic drinking water services, and nearly half of them live in Least Developed Countries (LDCs)^7^. Water-borne illnesses account for over 60% of infant mortality rates. In Pakistan's rural areas, around 90% of the population lacks access to clean drinking water^8^. The UNICEF research states that 12.6% of newborn deaths and 7% of fertility in Pakistan are connected to water-associated diseases, including as cholera, diarrhea, malaria, hepatitis, typhoid fever, dysentery, and giardiasis, and that between 20 and 40% of patients in the country’s hospitals suffer from these conditions. Every year in Pakistan, between 0.2 and 0.25 million children die from diarrhea. Every year, 10,000 people in Karachi die from kidney infections caused by contaminated drinking water^9^.

Industrial discharge can have a substantial impact on drinking water quality by bringing numerous pollutants into the environment, which in turn causes 82% of diseases like cholera, dysentery, and typhoid. Water bodies are primarily affected due to discharge of untreated or inadequately treated effluents that contain harmful pollutants such heavy metals, organic compounds, and excessive salt content. The safety and quality of drinking water resources are at stake because these pollutants have the ability to contaminate surface water and seep into groundwater sources. Inadequate management and elimination of industrial waste can result in enduring environmental deterioration and possible health risks for populations reliant on these water supplies^10^. An official survey conducted across 12 districts in Punjab indicated that around 79% of drinking water samples were contaminated, while 88% of drinking water in rural areas was contaminated due to sewage discharge, heavy metals, microorganisms, and industrial effluents^11,12^.

It is crucial to comprehend the spatial distribution of environmental features in order to evaluate the quality of drinking water. However, it can be expensive to monitor the water quality, particularly in big groundwater basins. Therefore, dependable and flexible gadgets will be required to solve such problems. Use of technologies like geographic information systems (GIS) facilitate spatial analysis, water quality monitoring, and support strategic planning and decision-making processes linked to water management. In the event of an emergency or outbreak of a waterborne disease, GIS can also help with real-time tracking, monitoring, and visualization of the impacted areas, the people at risk, and the resources that are available. GIS-generated interactive maps and visualizations can be used to make communities aware about water-related issues and to increase public understanding of potential hazards, sources, and quality of drinking water. Through the application of GIS technology, the drinking water quality of the entire region may be presented with fewer observations, which lowers costs and improves the overall effectiveness of water management and monitoring initiatives. Synthetic pollution index (SPI) and the water quality index (WQI) are among the most often used methods for classifying and reflecting the quality of the water and pollution risk in a given area. WQI and SPI have been utilized by researchers from several countries to evaluate the water quality in various places^13,14^.

The purpose of the present study was to evaluate the quality of the drinking water in the Gulshan-e-Ravi and Samanabad localities using the WQI and SPI. These areas are particularly ancient and are posing health risks including waterborne and microbial ailments like diarrhea and cholera, etc. Major reasons that lie behind the water pollution are industrial discharges, low groundwater levels, dumping, and old and worn-out metallic pipes. The study area i.e., Gulshan-e-Ravi, and some colonies of Samanabad zone has severe issue of water pollution. This can be observed in the form of common waterborn diseases rate. The key objectives of the present study are to identify the location points having high physico-chemical attributes by the GIS approach and to calculate the WQI and SPI of selected areas by determining the physico-chemical parameters in the water samples.

## Materials and methods

**Figure 1:**
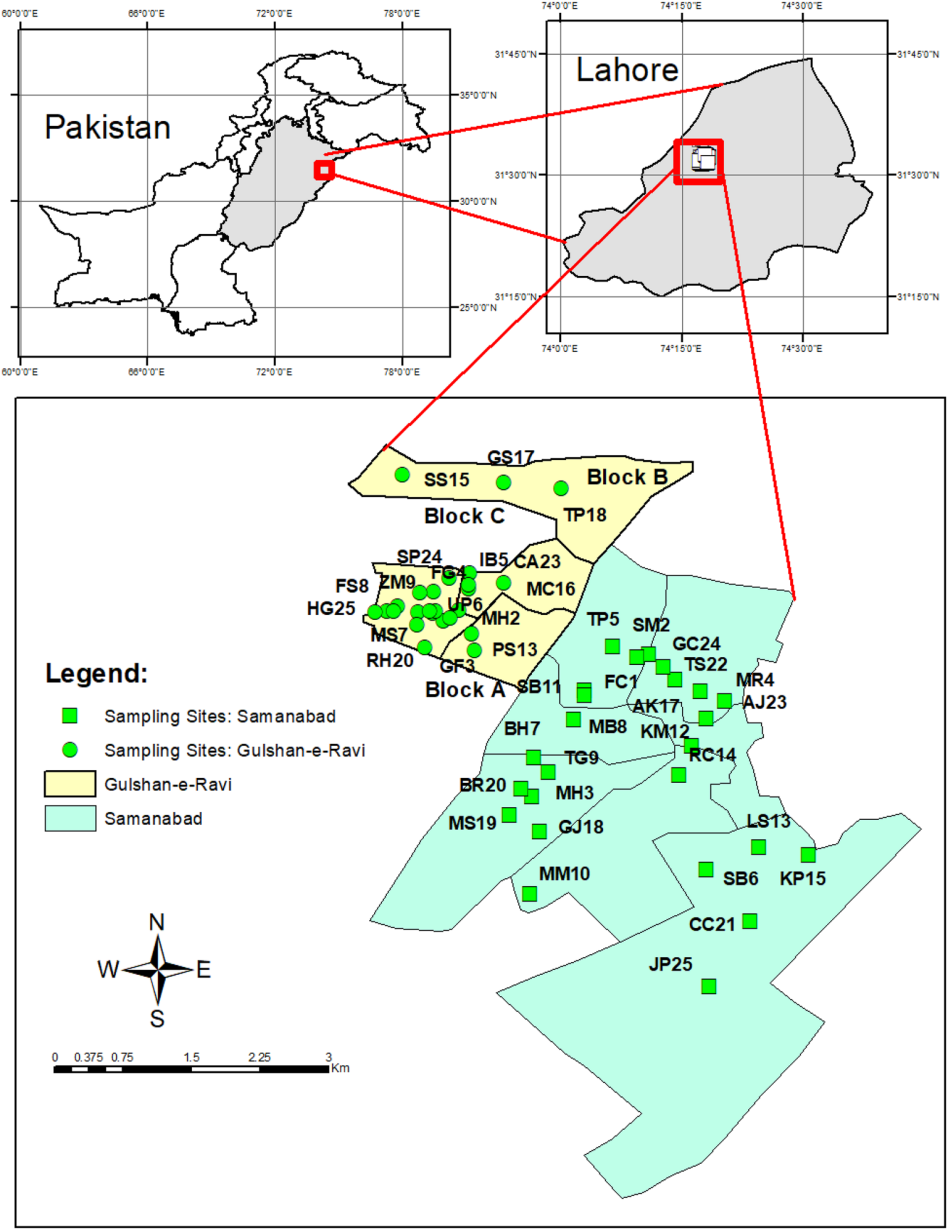
Map showing study areas with sampling sites.

The study was conducted in a densely populated city, Lahore, Punjab province, Pakistan. The longitude and latitude are 31.5204° N and 74.3587° E^15^. Study areas were Gulshan-e-Ravi and Samanabad town which are situated in the Samanabad zone of Lahore as shown in Fig. 1. Lahore is located on the northeast side of the country having an international border (Wahga Border) with India. The northern part of the city is considered a walled city (old city). It is the provincial capital of Punjab. Pakistan’s second most populated city is Lahore with a population of more than 13 million. It is the 26th most populated city in the world^16^. The climate of Lahore is comprised of five seasons. Pakistan is fortunate to have these distinct seasons: summer, winter, autmn, spring, and monsoon. These unique weather patterns and seasons are important to Pakistan’s geographical circumstances. The hottest month of Lahore is June having a 38.2 °C average temperature, while the coldest month is January having a 12 °C average temperature. In monsoon months, maximum rainfall is observed^17^. According to the 2017 census, Lahore’s population is 11,126,285. During the past decades, the inhabitants of Lahore have grown extensively.

### Hydrogeological setting

Lahore aquifer, a 400-m-thick unconsolidated alluvial complex in Bari Doab, is a highly transmissive, 25–70 m/day hydraulic conductor in the south-flowing Indus River system. It is reported that there are two aquifers in the Lahore area, the shallow and deep, separated by an aquitard^18^. Despite the heterogeneous composition of the alluvial complex, groundwater occurs under water table conditions^19^. Another study found that the soil in the Lahore area is predominately composed of quartz, muscovite, and clinochlore as major minerals with small percentages of heavy minerals and can be classified as silty clay forming part of Pleistocene deposits^20^. The River Ravi is a major recharge source and controls the overall hydrological flow in the study area. The area is generally flat, sloping slightly to the south and southwest direction with a gradient of 0.3–0.4 m/km.

With respect to land cover/land use of study areas, Gulshan-e-Ravi, a predominantly residential area in Lahore, offers a diverse range of housing options, including multi-story apartment complexes and large-yard homes. Gulshan-e-Ravi, situated on the Ravi River’s eastern bank is characterized by alluvial soils and sedimentary deposits, making it ideal for farming. Gulshan-e-Ravi’s hydrogeological characteristics are impacted by the Ravi River’s proximity. Groundwater is a major source of water for residents, primarily accessible through boreholes and tube wells. The most dominant land use in the study area is residential with a densly populated housing setting that changed rapidly between the years 2000 to 2005 and 2010 to 2015^21,22^. The local geological characteristics and the depth of the groundwater table can affect both the quantity and quality of the water. However, Samanabad, an older and established residential area in Lahore, is predominantly urban with a mixture of modern constructions and narrow streets. It is located on alluvial plains with sedimentary layers, has rich, ideal soil for agriculture, despite decreased agricultural land due to urbanization, and its water source comes from groundwater.

### Physico-chemical analysis

Drinking water samples were collected from 2 locations in Lahore i.e., Gulshan-e-Ravi and Samanabad, which are old urban areas of District Lahore, Pakistan. 25 water samples were collected from both areas. New plastic water bottles were used for sample collection to avoid any kind of contamination, along with proper care and labeling of bottles. Bottles were properly washed 2 to 3 times and dried before the sampling. Random sampling method was used to collect the water samples at various locations within the study areas during the months of August and September. Water quality parameters are significantly influenced by the monsoon season, characterized by heavy rainfall. This precipitation can lead to increased runoff from urban areas, potentially transporting pollutants into water bodies. Rising temperatures, agricultural practices, and the discharge of untreated wastewater further contribute to the complex interplay of factors affecting water quality during these months^23^.

A tap was run for 2–3 min before collecting a sample to help flush out stagnant water. The sample bottle was held below tap flow, filled to the specific line, and sealed. The sample bottle was then labled. Longitudes and latitudes were also recorded instantly. Numerous water quality parameters, including physical (temperature, turbidity, TDS) and chemical (pH, EC, DO, total hardness (TH), calcium (Ca^+2^) hardness, and magnesium (Mg^+2^) hardness) were determined in the laboratory. Temperature and pH were measured at the site of sample collection. All physico-chemical parameters were analyzed by following the standard methods of the American Public Health Organization (APHA) and the American Society for Testing and Materials (ASTM) as shown in Table 1. These guidelines were followed throughout the examination process. This commitment to established standards not only ensures the accuracy and reliability of the data but also facilitates comparability with existing research, contributing to a more robust and credible assessment of groundwater quality. ArcGIS (10.8) software was used, employing the interpolation technique, to develop spatial maps for identifying the areas with polluted drinking water.

**Table 1:** Analysis methods used for testing various parameters.

Sr. no	Parameters	Analysis method	Reference
1	pH	pH meter, pH-208	^24^
2	Temperature (°C)	Thermometer	
3	Dissolved oxygen (ppm)	Dissolved oxygen analyzer (DO1900)	
4	Total dissolved solids (ppm)	TDS meter (M1)	
5	Electrical conductivity (µS)	Conductivity meter (CD 4306)	
6	Turbidity (NTU)	Turbidity meter (TU-2016)	
7	Total hardness (mg/L)	EDTA titration, Erichrome Black-T indicator, Standard method (2017)	
8	Calcium (mg/L)	EDTA titration, murexide indicator, standard method (2017)	
9	Magnesium (mg/L)	EDTA titration, standard method (2017)	

Total hardness (TH), Ca^+2^ hardness, and Mg^+2^ hardness were analyzed using the standard EDTA titration method. For total hardness, an ammonia buffer solution was prepared and added to a 50-water sample. A pinch of Erichrome Black-T was added and suddenly the color of the sample changed from transparent to wine red. Then it was titrated against EDTA present in the burette, the colour changed to dark blue. Total hardness was calculated by observing the initial and final burette readings by the following formula;

1 $$Total\;EDTA\;used = Final\;Reading \left( {T2} \right) - Initital\;reading \left( {T1} \right)$$

2 $$Total\;Hardness = \frac{{Total\;EDTA\;used \left( {ml} \right) \times M \left( {0.0149} \right) \times 1000}}{{Volume\;of\;sample \left( {ml} \right)}}$$

For calcium (Ca^+2^) hardness, 2 ml of 1 M NaOH (sodium hydroxide) solution was prepared and a few drops were added to the 50 ml of water sample with the help of a pipette. Then after stirring, a pinch of murexide (C_8_H_3_N_5_O_6_^–2^) was added to the water sample. After a little stirring, the sample watercolor was changed to a pink color. Then, it was titrated against the EDTA solution present in the burette, and the color was changed from pink to purple indicating the presence of calcium. Calcium hardness was calculated by following the formula;

3 $$Total\;EDTA\;used = Final\;Reading \left( {T2} \right) - Initital\;reading \left( {T1} \right)$$

4 $$Calcium\;contents = \frac{{Total\,EDTA\;used \left( {ml} \right) \times 400.5 \times 1.05}}{{Volume\;of\;sample \left( {ml} \right)}}$$

Magnesium (Mg^+2^) hardness is calculated by the difference between total and calcium hardness by given following formula;

5 $$Magnesium\;hardness = Total\;Hardness - Calcium\;Hardness$$

### Water Quality Index (WQI)

The nine significant physico-chemical parameters were utilized for estimation of WQI from the study site to assess the quality of drinking water. WHO permissible values for drinking water were used to compare these parameters using the formula for calculating WQI^25^.

To analyze WQI, firstly relative weight (*W*_*i*_) was calculated using the given formula:

6 $$w_{i} = \frac{K}{{S_{i} }}$$

K was calculated using;

7 $$K = {1 \mathord{\left/ {\vphantom {1 {\left( {\frac{\sum 1}{{S_{i} }}} \right)}}} \right. \kern-0pt} {\left( {\frac{\sum 1}{{S_{i} }}} \right)}}$$

where, Wi is the unit weight factor, K is the proportional constant, Si is the standard permissible value of i^th^ parameter.

The unit weight for all the chosen nine parameters with their standard values was calculated. A number that reflects the relative value of the given parameter in the contaminated water referring to its permissible standard value is the quality rating scale (Qi) and it was calculated using the formula;

8 $${Q}_{i}=\frac{{V}_{i}}{{S}_{i}}\times 100$$

where, Q_i_ is the quality rating scale of i^th^ parameter, V_i_ is the estimated permissible value and S_i_ is standard permissible value of i^th^ parameter.

All the values of V_o_ were taken as 0 for the drinking water, except for pH and DO i.e., 7.0 and 40 ppm. After finding *w*_*i*_ and* q*_*i*_*,,* both values were multiplied with each other by having w_i_q_i_ and then it was divided with *w*_*i*_:

9 $$wiqi=wi\times qi$$

Then overall WQI was calculated;

10 $$WQI= \sum_{i=1}^{n}{w}_{i}\times {q}_{i}$$

WQI for water samples of both sites 1 and 2 was calculated using the Eq. (10). WQI generally ranges between good to poor category^26^. The water quality of the selected areas was classified into different categories using WQI, as given in Table 2.

**Table 2:** Analysis methods used for testing various parameters.

WQI	Water quality ratings
0–25	Excellent
26–50	Good
51–75	Poor
76–100	Very poor
> 100	Unfit, unsuitable for drinking

Source:^27^.

### Calculation of synthetic pollution index (SPI) model

The derivation and calculation of SPI involves different steps given below^28^:

Step 1: Constant of proportionality (*Ki*):

11 $$K = {1 \mathord{\left/ {\vphantom {1 {\left( {\frac{\sum 1}{{S_{i} }}} \right)}}} \right. \kern-0pt} {\left( {\frac{\sum 1}{{S_{i} }}} \right)}}$$

Step 2: Weight coefficient (*Wi*):

12 $$Wi=\frac{Ki}{Si}(i = 1, 2, 3, \dots \dots , n)$$

Step 3: Synthetic pollution index (SPI):

13 $$SPI={\sum }_{i=1}^{n}\frac{Ci}{Si}\times Wi(\text{i}=1, 2, 3\dots ..,\text{ n})$$

where, *Si* is the threshold value for an *i*^th^ physicochemical parameter as per WHO guidelines and *n* is the total number of water quality parameters considered for analysis. Based on SPI, the water quality is classified into five categories as shown in Table 3;

**Table 3:** Water quality classification based on SPI.

SPI	Water quality class
SPI < 0.2	Suitable
0.2 ≤ SPI < 0.5	slightly polluted
0.5 ≤ SPI < 1.0	moderately polluted
1.0 ≤ SPI	highly polluted
SPI ≥ 3.0	unsuitable for drinking purposes

Source:^28^.

## Results and discussion

Results from the present study revealed the significant variations in different physico-chemical parameters of sampling sites. Some of the water samples had paramters’ values below and some of them had above the WHO guidelines for drinking water.

### Analysis based on physico-chemical parameters

**Figure 2:**
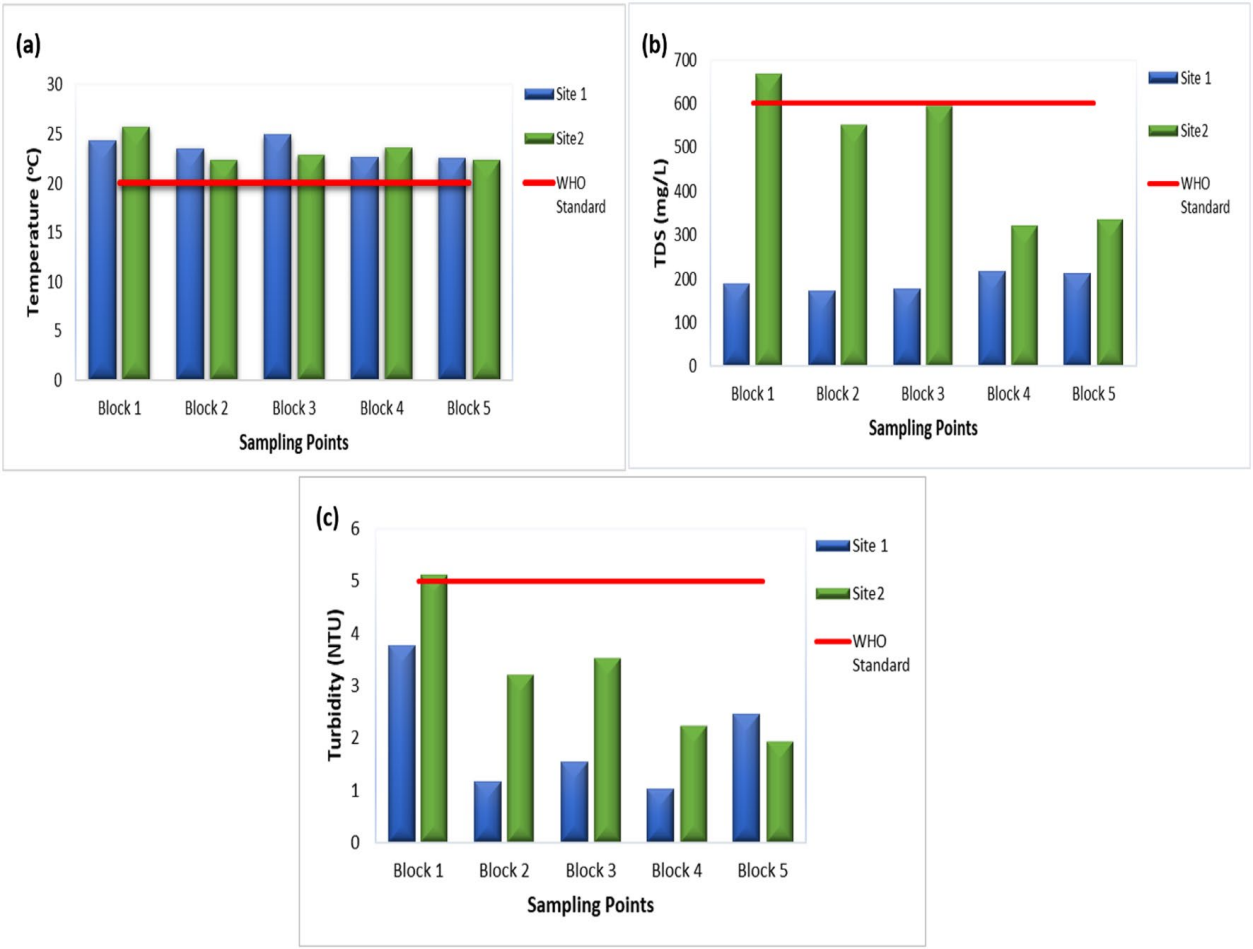
Concentration of physical parameters: Temperature (**a**), TDS (**b**), and Turbidity (**c**) in drinking water of selected areas of Lahore.

The pH level of a solution indicates its alkalinity or acidity, determined by the concentration of hydrogen ions within the solution. Typically, the pH scale varies from 0 to 14. At 25 °C, the acidic aqueous solutions have a pH under 7. While basic or alkaline aqueous solutions have a pH above 7. Furthermore, a pH level of 7 at 25 °C is considered as “neutral”. As the H_3_O^+^ ions concentration becomes equal to the OH^–^ ions concentration in pure water. Strong bases may have a pH above 7 to 14, while strong acids have a pH of less than 7 to 0^29^. WHO guideline for pH of drinking water is 6.5 to 8.5. In this study, most of the water samples had pH between the range, as shown in Fig. 2. Block 1 of site 1 (Gulshan-e-Ravi) average pH was slightly below the WHO guideline i.e., 5.87. While, Block 1 and 3 of site 2 (Samanabad) average pH was slightly above the guideline i.e., 8.54 and 8.66. Some of the areas in Blocks 1 and 3 had water contamination issues due to old and corrosive water pipelines. The blue color in the pH map indicates high pH values (alkaline) exceeding the WHO guidelines in the study sites figure. Most of the problem lies in acidic water compared to basic water which causes skin issues. Also, the human kidney system is considered to be the best filtration system to maintain the acid–base situation in the human body. And alkaline water has an advantage in improving gut health and lowering blood sugar levels^30^.

The temperature of the water is also important physical parameter for assessing water quality. Temperature can affect many other factors as well and it can alter chemical and physical properties of water^31^.

According to WHO, the standard temperature for drinking water should be between 20 and 25 °C. Both study sites had temperatures within range except for Block 3 of Site 1 and Block 1 of Site 2. Both values were slightly above the guideline and this might be mainly due to the sample collection season. The temperature map shows variations in the temperature of both study sites. The most significant temperature fluctuations are depicted in yellow (22–24 °C), followed by orange and then green. Areas exceeding WHO guidelines are presented in red color. High temperatures may increase the microbial activity and this can affect other parameters such as pH and electrical conductivity.

The TDS consists of inorganic salts including Ca^+2^, Cl^−^, K+, Na+, Mg^+2^, HCO_3_^−1^, and SO_4_^−2^ and a few other small amounts of such organic contents, minerals or metals which are dissolved in a specific amount of water^32^. Higher levels of TDS affect the drinking water quality. According to a study^33^, TDS in drinking water shouldn’t be more than 500 mg/L or ppm. If it exceeds more than 600 or 1000 mg/L, it is not considered fit for drinking. TDS are mostly increased by industrial sewage, rocks, urban runoff, silt, and the use of fertilizers and pesticides. WHO guideline for TDS in drinking water is 600 mg/L. Site 1 samples had TDS within the WHO guideline with an average maximum value of 192.5 mg/L. While site 2 had serious issues regarding TDS in drinking water. Drinking water in 4 out of 5 blocks had TDS higher than the WHO guideline, as shown in Fig. 2. The average highest value of TDS was 779 mg/L in Block 1 of site 2. While, 80% of site 2 water samples had TDS higher than guidelines, exceeding 1000 mg/L.

**Figure 3:**
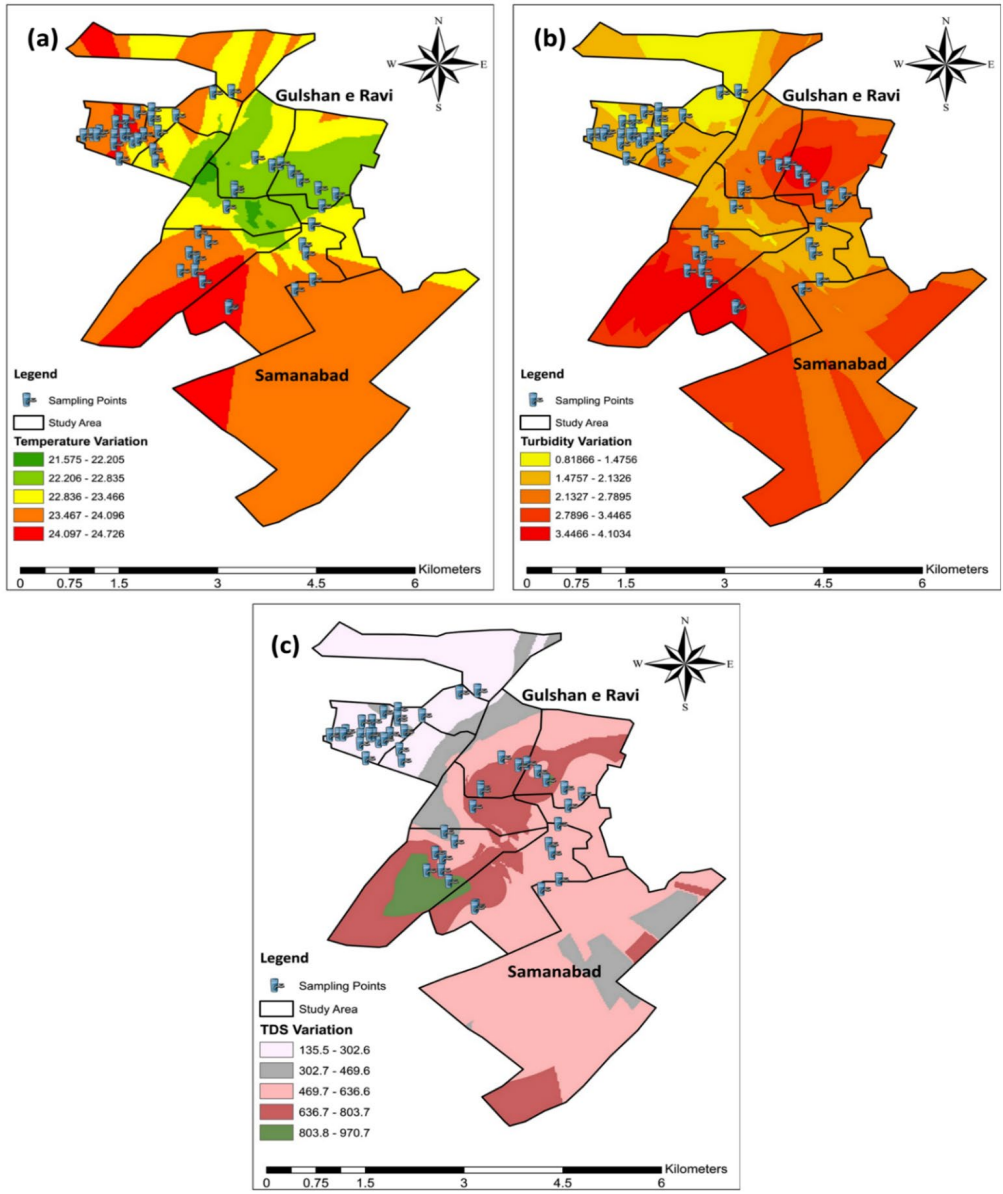
Water quality status map for Gulshan Ravi (Site 1) and Samanabad (Site 2): Temperature (**a**), Turbidity (**b**), and TDS (**c**) are visualized through a GIS-based map, illustrating the water quality conditions at both sites.

The management of groundwater for domestic and agricultural consumption requires a thorough qualitative assessment and a comprehensive understanding of spatial variation^34–36^. For this purpose, spatial distribution maps were also incorporated into this study as shown in Fig. 3. The map of chemical parameters such as TDS indicates high TDS values exceeding WHO standards in Site 2 (yellow, orange, and red). The most of the water samples had TDS level between 472 and 672 mg/L in site 2 while, Site 1 had a TDS level within the permissible range of WHO standards which is indicated by blue color (137–305 mg/L), as shown in Tables 4 and 5. High levels of TDS in drinking water and domestic use can lead to nausea, vomiting, dizziness, lung irritation, and rashes. While long term usage of such water can cause chronic health issues such as liver and kidney failures, cancer, weak immunity, nervous system disorders, and birth defects in newborn babies. Pakistan is facing health risks due to poor monitoring and maintenance, ranking as one of South Asia’s most water-polluted countries with urban areas contributing to increasing health and environmental issues^37,38^.

**Table 4:** Summary of various drinking water physiochemical parameters of study site 1.

Parameters	pH	Temperature (°C)	Turbidity (NTU)	DO (mg/L)	EC (µS/cm)	TDS (mg/L)	TH (mg/L)	Ca (mg/L)	Mg (mg/L)
Permissible Range	8.5	25.43	5	8	800	500	500	120	50
Minimum	5.87	22.52	0.95	6.58	429	170.4	67.6	18.72	24.22
Maximum	8.6	24.88	1.27	7.14	814.4	216	196.2	34.88	40.4
Average	7.37	23.57	1.11	6.90	716.88	192.56	126.84	25.04	29.47

**Table 5:** Summary of various drinking water physiochemical parameters of study site 2.

Parameters	pH	Temperature (°C)	Turbidity (NTU)	DO (mg/L)	EC (µS/cm)	TDS (mg/L)	TH (mg/L)	Ca (mg/L)	Mg (mg/L)
Permissible Range	8.5	25	5	8	800	500	500	120	50
Minimum	7.26	22.4	1.92	6.76	722.6	524	170.4	22.16	27.28
Maximum	8.84	25.72	5.1	7.54	877.6	779.2	514.8	123.42	53.1
Average	8.12	23.40	2.79	7.15	815.44	621.96	343.92	65.73	39.52

Turbidity is caused by suspended waterborne particles, including fine inorganic or organic substances, sediment, and microscopic organisms like algae, scattering of light, and cloudy or opaque appearance of water. These particles can consist of fine sediments like silt or clay, and various others. A low level of turbidity indicates high clarity of water, while a high level of turbidity indicates low clarity of water^39^. According to a study^40^, drinking water turbidity should be less than 5 NTU (Nephelometric Turbidity Unit). High levels of turbidity may not seem aesthetically clean and water is not fit for drinking purpose. Most of the samples were within the permissible range recommended by WHO that indicated that the water in these sites was clear. One case was detected in block 1 of site 2, which had a slight 1% high turbidity in water, still, it made the water cloudy. The highest turbidity issue was reported in both sites but especially in site 2 have a turbidity of more than 5 NTU. High turbidity can hinder disinfection issues in the water and it can lead to high growth of microorganisms such as parasites, bacteria, and viruses. Drinking water with high turbidity can cause nausea, diarrhea, cramps, and headaches especially in infants, as they are more prone to diseases. Other than those, the elderly and weak immunity people can also be affected by such problems. Also, it seems poor aesthetically^33^ and people boil water before use in case of high turbidity of water.

A greater EC indicates that the groundwater is more enriched in the salts. For dissolved ionizable solid (Na, Ca, and Mg salts) concentrations and salinity, EC works as an indicator. Due to the effect of anthropogenic activities, more pollutants move into groundwater and hence, EC increases^41,42^. It is measured in micro-Siemens per centimeters (µS/cm) or milli-Siemens per centimeters (mS/cm). i.e., 1 mS = 1000 µS and 1 µS = 0.001 mS. WHO guideline for EC in drinking water rages from 200 to 800 µS/cm, while 800 µS/cm is the MPL for drinking water. Block 2 (849.8), 3 (814.4), and 5 (844.6) of site 1 had more EC than the standard. While in site 2, block 1 (877.6), 3 (897), and 4 (818) had high EC specifically block 3 had the highest one followed by block 2 of site 2 and then block 2 and 5 of site 1. The highest EC (> 800 µS/cm) was recorded in both sites which is indicated by the white and tea-pink color. High EC causes high corrosiveness in the water. EC has no direct health link but it can lead to other fluctuations in parameters like pH, total hardness, and TDS, which can cause minerals like the taste of water and health issues like skin problems and gut problems.

Dissolved oxygen (DO), necessary for aquatic life, can be negatively affected by the presence of organic material, agricultural runoff and leaching, industrial waste, and dissolved gases, with concentrations below 5.0 mg/L^32^. Sufficient DO is essential for water quality, higher levels of DO affect aquatic life and potentially corrode water pipes, while low levels indicate increased microbial activity. WHO guideline for drinking water DO is 6.5 to 8 mg/L or ppm. Almost every sample was within the range of standard. The permissible value of DO in water indicates that oxygen concentration is fine for drinking purposes. DO concentration of both sites was within the range of WHO guideline except for 2 to 3 sampling points of site 2. The reason might be some anthropogenic factors which increase temperature and hence microbial activity starts.

Total hardness indictaes the magnesium (Mg) and calcium (Ca) dissolved in the water, to measure the solubility of water for drinking purposes, local households, and some industrial applications credited to the occurrence of Ca^+2^, Mg^2+^, Cl^−^, HCO_3_^−1^, and SO_4_^−2^. Specifically, alkaline earth metals Ca and Mg in dissolved form, play an important role in water hardness. It is measured in milligrams per liter (mg/L) of calcium carbonates by combining overall contents^32^.

Water having hardness below 75 mg/L is soft water, followed by 76 to 150 mg/L lies in moderately hard water, 151–300 mg/L is categorized as hard water and more than 300 mg/L is considered very hard water^43^. WHO guidelines for total hardness should be no more than 500 mg/L in drinking water. Block 5 of site 2 had the highest water hardness recorded in the study area. Overall, site 2 water samples were mostly in the very hard water category and in contrast with site 1, most of the samples were soft water and moderately hard. This indicates that site 1 samples were in the permissible range of WHO having good water quality while site 2 had hard water issue due to densely populated areas and old scaly waterpipes. According to a study^44^, effective management strategies are required to prevent groundwater contamination and pollution, primarily in monitoring wells, and ensure daily access to alternative water sources for the local population.

The majority of water hardness was observed in site 2 having light and dark pink colors. While site 1 had total hardness within range and was shown with light and dark blue color. Although water hardness is not a health concern, it can cause problems in the home while washing clothes, dishwashing, bathing, and making clothes stiff and rough. Sticky soap curd is formed when soap is utilized with hard water, this can cause hurdles while cleaning. Also, it causes psychological issues may happen when this type of situation happens. While bathing, when the soap curd sticks with the body it prevents the removal of bacteria or dirt from the body and this can cause irritation and allergic itching problems in humans. In addition, water hardness reduces water flow in pipelines and hence Ca^+2^ and Mg^2+^ deposits in the pipelines ultimately require pipe replacement^45^.

**Figure 4:**
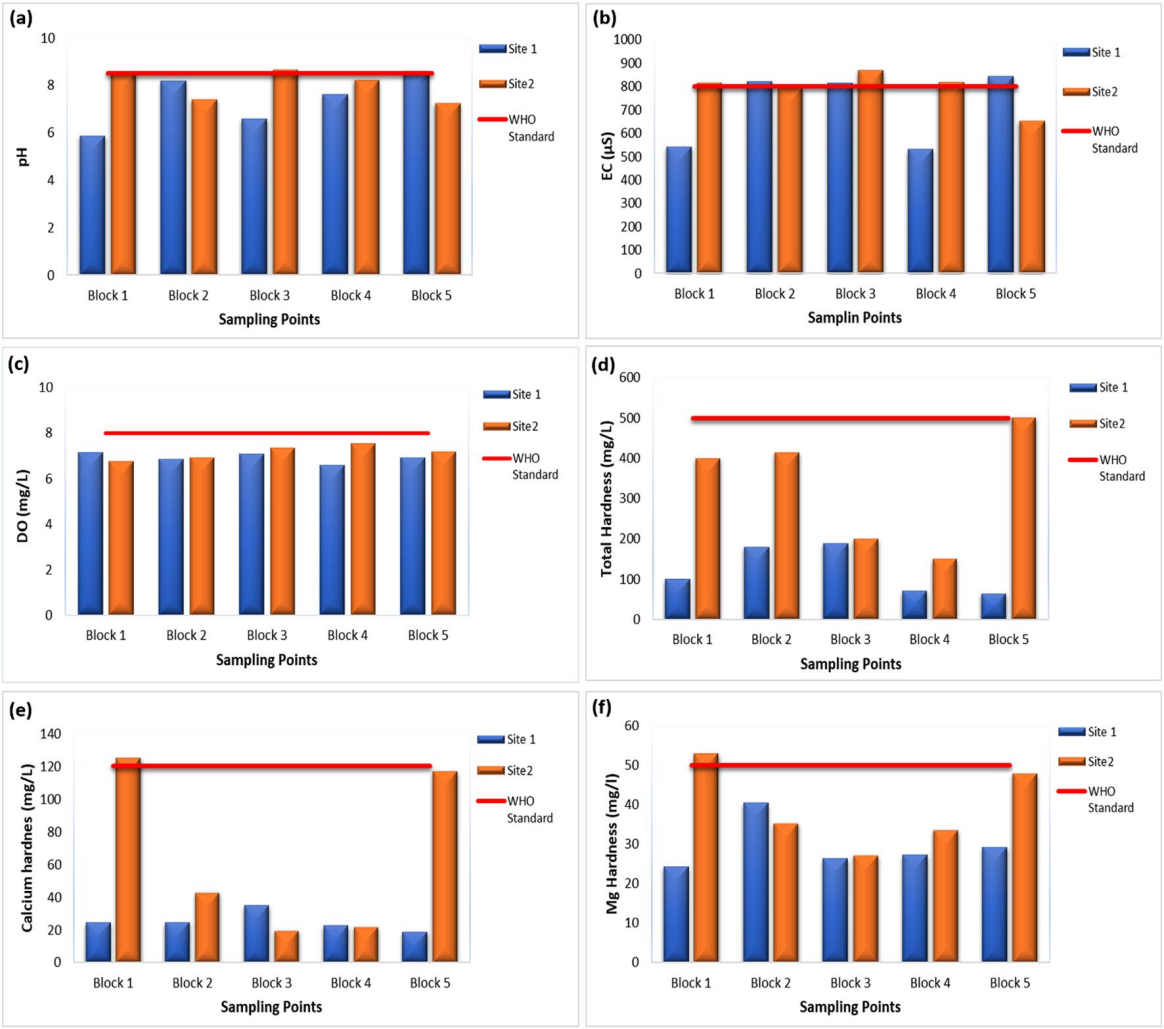
Concentration of selected chemical parameters: pH (**a**), EC (**b**), DO (**c**), Total Hardness (**d**), Calcium hardness (**e**), and Mg hardness (**f**) in drinking water of selected sites of Lahore.

**Figure 5:**
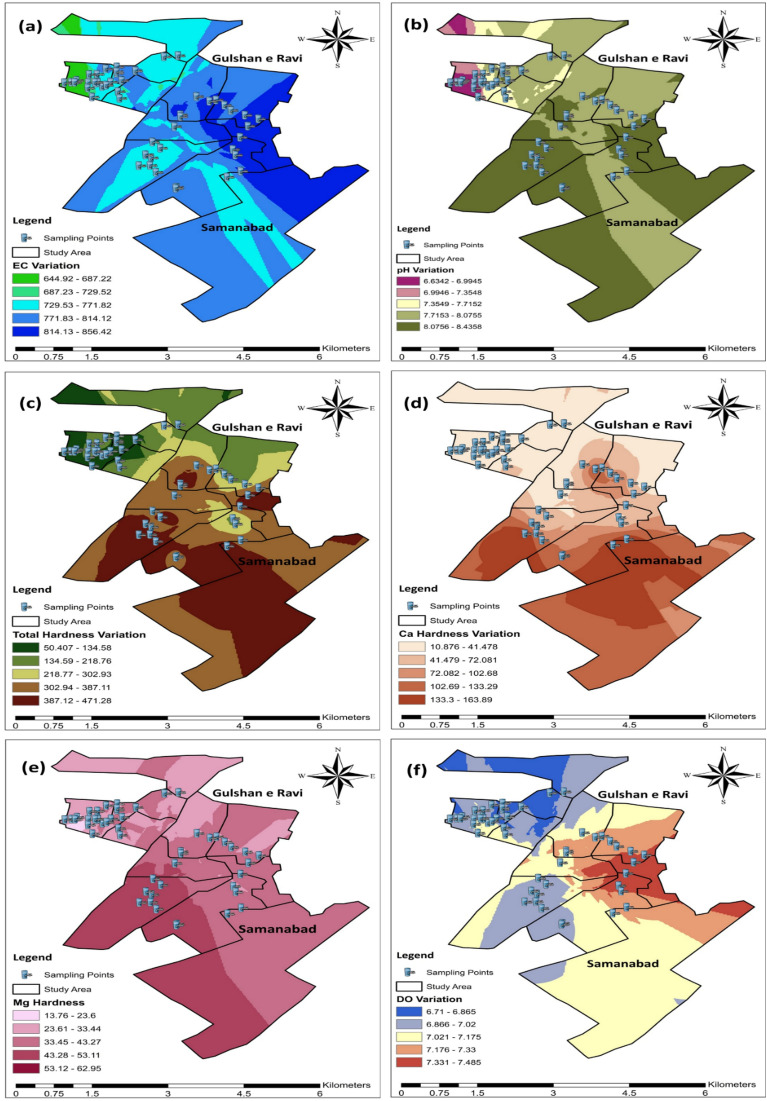
Water quality status map for Gulshan Ravi (Site 1) and Samanabad (Site 2). EC (**a**), pH (**b**) and TH (**c**), Calcium hardness (**d**), Mg hardness (**e**) and DO (**f**) are presented using a GIS map to illustrate the water quality conditions at both sites 1 and 2.

The amount of dissolved calcium in the water is represented in mg/L or ppm (parts per million) of calcium carbonates. Limestone is the major source of Ca hardness in water. Also, calcium can react with Fe, Zn, P, and Mg while reducing the absorption of other minerals. WHO standard for Ca^2+^ contents or hardness in drinking water is 60 to 120 mg/L. Whereas, 120 mg/L is the MPL for any drinking water. Ca^2+^ contents in current study areas varied depending upon the location. Block 1 and 5 of site 2 had the highest Ca^2+^ contents i.e., 123 and 118 mg/L, which exceeded the standard of WHO. Site 1 of the study area had Ca^2+^ contents within the permissible range and the water was considered as soft as shown in Fig. 5. Moderately high Ca hardness was observed in some areas of Site 2 (shown with dark blue color). While most of the water samples of both study sites were within range as shown in Fig. 4. High Ca^2+^ contents can weaken the bones; forms kidney stones and it also interfere with our brain and heart working. All of these problems can lead to hypercalcemia.

The presence of high Mg^2+^ in the form of SO_4_^−2^ and CO_3_^−2^ in drinking water is magnesium hardness. It is measured in mg/L or ppm. Dolomite is the major cause of magnesium hardness in water^46^. The WHO standard for Mg^2+^ concentration in water is 50 mg/L. Most of the values in the current study site were in the permissible range, but a few like block 1 of site 1 and block 5 of site two had slightly higher Mg^2+^ content in water as shown in Fig. 4. This indicates that Mg^2+^ deposits were present in the pipelines due to high sewage content. The map indicates a magnesium hardness trend in both areas. Some areas with dark purple values had high Mg hardness as compared to others with light colors. High Mg^2+^ can cause hypermagnesemia, which causes renal failure resulting in the reduced ability to remove magnesium from the kidney. Bowel functions can also be disturbed by high Mg^2+^ contents. The local hydrological setting controls water movement in the south and southwest directions towards the Ravi River. Soil–water contact enhanced the dissolution of minerals, enriching water with sodium and calcium concentration (Fig. 5), resulting in the rise of total dissolved salts. Mineral–water contact time brings salt concentration (~ 1000 mg/L) levels that render it unsuitable for some uses.

### Analysis based on the WQI model

The Water Quality Index maps were developed using ArcGIS software (10.8) on the basis of selective physico-chemical parameters, classified as excellent, very good, good, poor, and very poor^47^ as mentioned in Table 2. The factors affecting the water quality include all the physico-chemical parameters that were used to examine the water quality. These factors play a key role in identifying the water quality of an area^48^. Basically, this study includes the determination of physiochemical parameters of drinking water in current study sites and their water quality parameters, as shown in Table 5. Based on water quality factors, the WQI produces a single value that indicates the total water quality in a specific area. This is a composite indicator that combines the effects of many water quality parameters and suitability for drinking purposes^1,49,50^.

**Figure 6:**
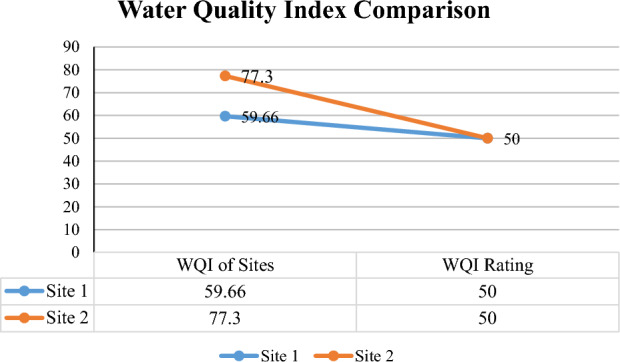
Comparison of average WQI for Gulshan Ravi (Site 1) and Samanabad (Site 2).

**Figure 7:**
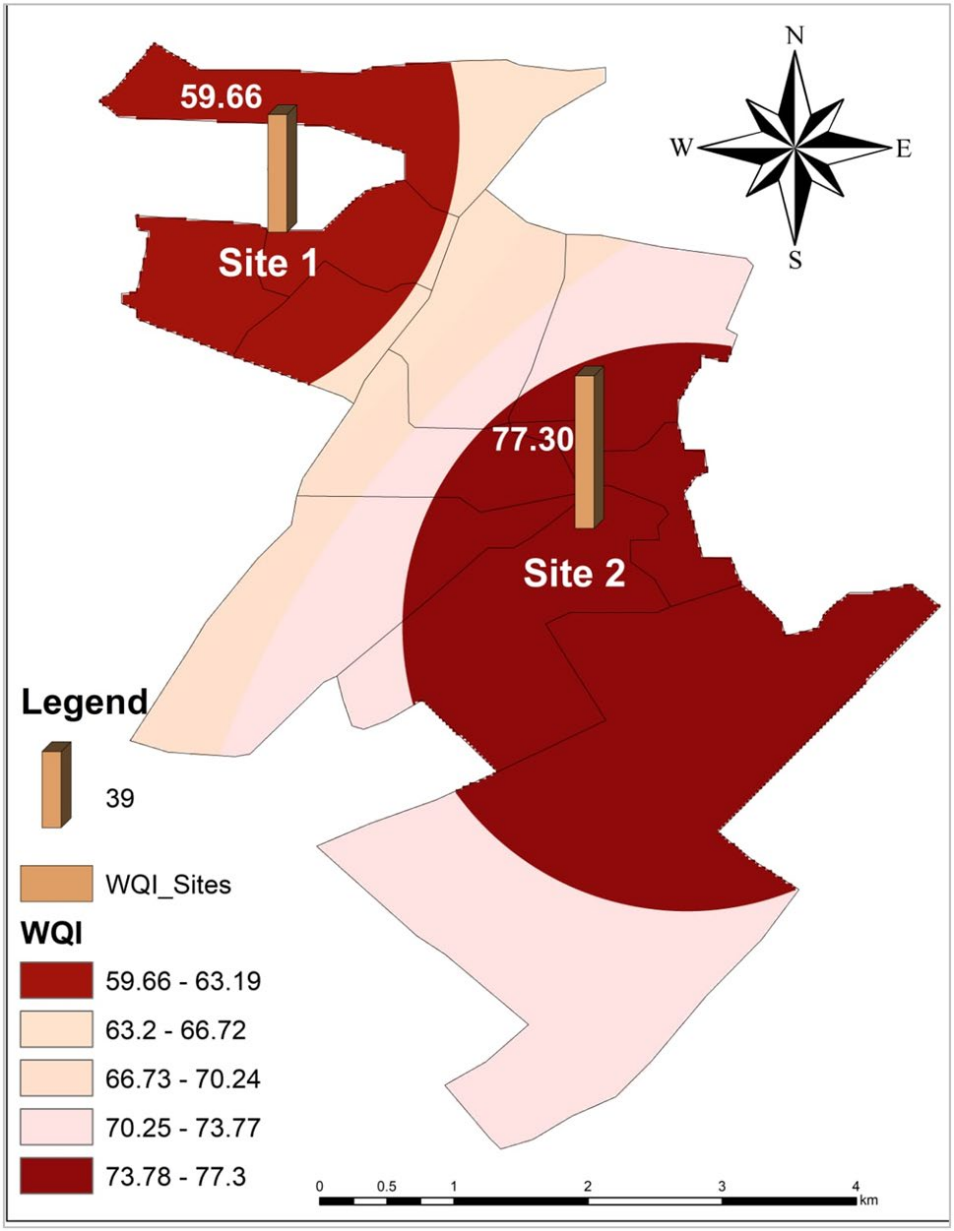
Spatial distribution map showing WQI of both site 1 Gulshan Ravi and site 2 Samanabad.

The WQI is a statistical tool that simplifies the analysis of complex groundwater data^51^. WQI of site 1 and site 2 was 59.66 and 77.3, respectively. Site 1 WQI lies in a “Poor” rating of water quality. Site 2 WQI lies in the “Very Poor” rating of WQI as shown in Fig. 6 which indicates that both areas either had some physiochemical parameters within range, but overall, the water quality rating is very poor and it poses a serious health threat to the residents of these areas. A comparative difference between both sites with the help of an interpolation map shows both areas were shown with dark colors which indicate poor and very poor water quality (Fig. 7). Findings of the current study regarding WQI are in line with a study conducted by^52^ to check water quality in western Lahore which has poor WQI and is unfit for human consumption. The observed differences in water quality, ranging from poor to very poor, can be attributed to a myriad of factors. Conversely, areas with poorer water quality experience contamination from industrial discharges, low groundwater levels, dumping, and old and worn-out metallic pipes. To address these disparities and improve water quality in deteriorating areas like Samanabad and Gulshan Ravi in Lahore, comprehensive mitigation measures are essential like water monitoring, and community awareness on responsible water usage are potential interventions. Additionally, strategic urban planning and infrastructure development can play a pivotal role in preventing further degradation and fostering long-term improvements in water quality.

### Analysis based on the SPI Model

The findings of the analysis of water samples for the purpose of determining the quality of drinking water and classifying it using SPI are compiled in Table 3.

Based on the SPI model, water samples of both areas were identified as “very polluted” as the SPI value was more than 3 indicating that there is a high risk of contamination of drinking water in these areas.

Water quality issues prevailing in the study area are similar to those found in other big urban areas of Pakistan^53^. SPI of water samples collected from a selected areas of Karachi varied from 0.6 to 6.6 and no water sample was found to be suitable for drinking purposes.

### The relationship between WQI and SPI models

**Figure 8:**
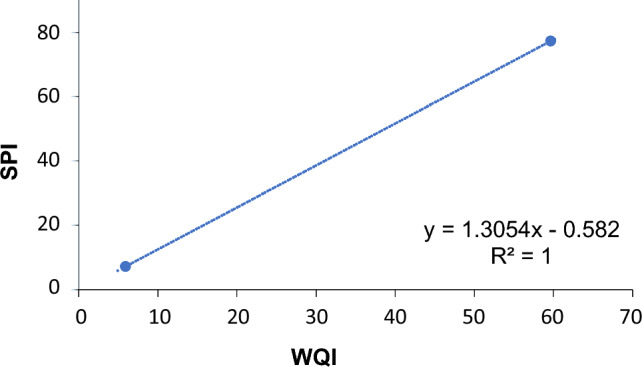
Regression analysis of Water Quality Index and Synthetic Pollution Index.

The respective WQI and SPI model categories of water were correlated using regression analysis in order to determine a relationship between them. The relationship shows a strong correlation between both models showing the R^2^ value is 1, as shown in Fig. 8. A series of studies have demonstrated a strong regression analysis between water quality index (WQI) and synthetic pollution index (SPI) in drinking water quality. Other study found a significant positive correlation between WQI and SPI, indicating an increase in pollution load^54^. This was further supported by another research study^55^, that reported a fair correlation between the two indices in the lower stretch of river Ganga. The threat of heavy metal pollution in drinking water, with a significant impact from Pb contamination are explored^56^. A study further improved the prediction of WQI using machine learning regression models, with linear regression and ridge offering the best performance^57^. These studies collectively underscore the importance of monitoring and addressing synthetic pollution in drinking water.

## Conclusion

The aim of the present study was to assess the quality of drinking water in two urban areas of Lahore using physico-chemical analysis, Water Quality Index (WQI), and the Specific Pollution Index (SPI). The findings revealed that in site 1 (Samanabad) had significant issues related to water quality, affecting primarily major residential colonies and blocks with elevated physico-chemical parameters suh as TDS, temperature pH, Ca^+2^, Mg^+2^, turbidity, etc. The major reasons for poor water quality are old water pipelines, rapid urbanization, toxic ingredients seepage, improper waste disposal, and low groundwater levels in these areas. Major parameters recorded that were above the WHO guidelines were EC, pH, TDS, and hardness. Although some parameters of both areas were within range as prescribed by WHO guidelines, WQI indicated that both areas had overall poor (59.66) and very poor (77.30) water quality ratings. WQI describes a greater number of variables using a single value that indicates the overall quality of water in a certain area. It is concluded that the water quality in both study areas was found unsuitable for drinking, emphasizing the need for prompt action by local authorities. Stricter management of industrial effluent, public education campaigns about water conservation, and the search for alternative water supplies should be the top priorities for remedial action. To reduce the risk of pollution, there is need of maintaining and modernizing sewage infrastructure, distribution networks, and provision of water treatment plants.

## Data Availability

All data generated or analyzed during this study are included in this published article.
