# Author Correction: Assessment of drinking water quality using Water Quality Index and synthetic pollution index in urban areas of mega city Lahore: a GIS-based approach

**DOI:** 10.1038/s41598-024-68537-x

**Published:** 2024-07-31

**Authors:** Maria Latif, Nimra Nasir, Rab Nawaz, Iqra Nasim, Khawar Sultan, Muhammad Atif Irshad, Ali Irfan, Turki M. Dawoud, Youssouf Ali Younous, Zulkifl Ahmed, Mohammed Bourhia

**Affiliations:** 1 Department of Environmental Sciences, The University of Lahore, Lahore, 54000 Pakistan; 2 Faculty of Engineering and Quantity Surveying, INTI International University, 71800 Nilai, Negeri Sembilan Malaysia; 3 Department of Chemistry, Government College University Faisalabad, Faisalabad, 38000 Pakistan; 4 Botany and Microbiology Department, College of Science, King Saud University, P.O. Box 2455, 11451 Riyadh, Saudi Arabia; 5 Evangelical College, BP 1200, N’Djamena, Chad; 6 College of Resource and Civil Engineering, Northeast University, Shenyang, China; 7 Laboratory of Biotechnology and Natural Resources Valorization, Faculty of Sciences, Ibn Zohr University, 80060 Agadir, Morocco

Correction to: *Scientific Reports* 10.1038/s41598-024-63296-1, published online 11 June 2024

The original version of this Article contained an error in the spelling of the author Zulkifl Ahmed, which was incorrectly given as Zulkfil Ahmed.

The original Article has been corrected.

